# Fast evolutionary rates associated with functional loss in class I glucose transporters of *Schistosoma mansoni*

**DOI:** 10.1186/s12864-015-2144-6

**Published:** 2015-11-19

**Authors:** Alejandro Cabezas-Cruz, James J. Valdés, Julien Lancelot, Raymond J. Pierce

**Affiliations:** Univ. Lille, CNRS, Inserm, CHU Lille, Institut Pasteur de Lille, U1019 - UMR 8204 - CIIL - Centre d’Infection et d’Immunité de Lille, F-59000 Lille, France; Institute of Parasitology, Biology Centre of the Academy of Sciences of the Czech Republic, 37005 České Budějovice, Czech Republic

**Keywords:** *Schistosoma mansoni*, Glucose transporters, Transcriptional regulation, Phylogeny, Biophysics

## Abstract

**Background:**

The trematode parasite, *Schistosoma mansoni*, has evolved to switch from oxidative phosphorylation to glycolysis in the presence of glucose immediately after invading the human host. This metabolic switch is dependent on extracellular glucose concentration. Four glucose transporters are encoded in the genome of *S. mansoni*, however, only two were shown to facilitate glucose diffusion.

**Results:**

By modeling the phase of human host infection, we showed that transporter transcript expression profiles of recently transformed schistosomula have two opposing responses to increased glucose concentrations. Concurring with the transcription profiles, our phylogenetic analyses revealed that *S. mansoni* glucose transporters belong to two separate clusters, one associated with class I glucose transporters from vertebrates and insects, and the other specific to parasitic Platyhelminthes. To study the evolutionary paths of both groups and their functional implications, we determined evolutionary rates, relative divergence times, genomic organization and performed structural analyses with the protein sequences. We finally used the modelled structures of the *S. mansoni* glucose transporters to biophysically (i) analyze the dynamics of key residues during glucose binding, (ii) test glucose stability within the active site, and (iii) demonstrate glucose diffusion. The two *S. mansoni* Platyhelminthes-specific glucose transporters, which seem to be younger than the other two, exhibit slower rates of molecular evolution, are encoded by intron-poor genes, and transport glucose. Interestingly, our molecular dynamic analyses suggest that *S. mansoni* class I glucose transporters are not able to transport glucose.

**Conclusions:**

The glucose transporter family in *S. mansoni* exhibit different evolutionary histories. Our results suggested that *S. mansoni* class I glucose transporters lost their capacity to transport glucose and that this function evolved independently in the Platyhelminthes-specific glucose transporters. Finally, taking into account the differences in the dynamics of glucose transport of the Platyhelminthes-specific transporters of *S. mansoni* compared to that of humans, we conclude that *S. mansoni* glucose transporters may be targets for rationally designed drugs against schistosomiasis.

**Electronic supplementary material:**

The online version of this article (doi:10.1186/s12864-015-2144-6) contains supplementary material, which is available to authorized users.

## Background

Upon contact with host mammalian skin, the free-living cercaria of *Schistosoma mansoni* undergoes dramatic biological [1] and metabolic transformations [2]. The metabolic transformation is a switch from an oxidative metabolism to a glycolytic metabolism that is reversibly dependent on external glucose concentrations [2]. In the mammalian host, glucose transport is facilitated by diffusion through the *S. mansoni* tegument [3]. Two closely related (61 % identity) schistosome glucose transporter proteins (SGTPs) have been functionally characterized in *S. mansoni*, named SGTP1 and SGTP4 [4, 5]. Both show stereospecificity for glucose, relaxed specificity for different hexoses and sodium-independent activity [4]. Differential protein expression profiles show that SGTP4 is expressed in the host-interactive outer tegument [6], while SGTP1 is mainly located in the basal membranes of the tegument [7]. Furthermore, Western blot analysis showed that SGTP1 is expressed in the egg, sporocyst, cercaria, schistosomula, and in adult male and female worms, but SGTP4 is only detected in mammalian-stage parasites (schistosomula and adults) [6]. Studies using RNAi showed that both SGTP1 and SGTP4 were critical for exogenous glucose uptake in schistosomula *in vitro* and for parasite development in vivo [5].

A third glucose transporter gene, *sgtp2*, has also been identified. The *sgtp2* open reading frame was interrupted by a missing base ~260 bp downstream the first initiator ATG codon. The hypothetical addition of a base at this downstream position resulted in a translation product homologous to glucose transporters [4]. Functional assays using *Xenopus* oocytes, however, showed that this hypothetical SGTP2 does not transport glucose (at 1 mM and 10 mM of substrate). In addition, transcripts of the *sgtp2* gene were only found in adult females [4], possibly due to a specific biological function at this stage. Although expressed sequence tags exist for all *S. mansoni sgtp* genes [5, 8], a fourth glucose transporter, *sgtp3*, is rarely mentioned in the literature.

The human glucose transporter protein family is divided into three classes: class I comprises GLUT1 to GLUT4 and GLUT14; class II comprises GLUT5, GLUT7, GLUT9, and GLUT11; and, class III comprises GLUT6, GLUT8, GLUT10 and GLUT12 (Augustin [9]). These three human glucose transporter classes are phylogenetically differentiated and show distinct molecular properties [9]. Among the human glucose transporters, GLUT1 and GLUT4 have been well studied and are transcriptionally upregulated under glucose deprivation conditions [10]. In terms of primary amino acid sequence, SGTP1 and SGTP4 were reported to be homologous to human GLUT1 [11], while SGTP2 is more similar to GLUT4 [4].

Glucose transporters undergo conformational shifts during glucose diffusion [12]. The resolved crystal structure of the proton symporter, XylE, of *Escherichia coli* has a glucose-bound, occluded structural conformation towards the intracellular compartment [13]. This occluded conformation disallows glucose diffusion into the cell. The crystal structure of human GLUT1, possesses a glucose-bound, open-inward structural conformation permitting glucose diffusion within the cell [12]. Comparing these two homologous transporter structures, Deng et al. [12] were able to hypothesize four intermediate conformational shifts during glucose binding and diffusion. XylE and GLUT1 represent the two respective, intermediate and sequential, glucose-bound conformations. The other two are glucose-free that represent conformations before glucose binding and after glucose diffusion [12].

In this study, the evolution of *S. mansoni* glucose transporters is explored and the effects of glucose-induced transcriptional regulation of the encoded genes were determined. Our findings led us to investigate glucose stability and migration with *S. mansoni* glucose transporters using a publicly available, state-of-the-art algorithm. These results provide insights into the molecular properties, evolution and biophysics of glucose transport in trematodes. Unveiling the structural and dynamic differences in glucose transport by parasitic worms can establish the basis for the rational design of schistosome-specific glucose transporter inhibitors.

## Results and discussion

### Glucose induces transcriptional changes in *S. mansoni* glucose transporter genes in schistosomula larvae

Regulation of nutrient transporters by nutrient availability is a well-known phenomenon in microorganisms, such as yeast [14] and bacteria [15]. The regulation of mammalian transporters by their substrates, however, is less understood [10]. Nevertheless, the effect of glucose on the transcriptional regulation of glucose transporters has been studied both *in vitro* and in vivo [10]. From these studies it is clear that mRNA levels of class I glucose transporters *glut1* and *glut4*, the most extensively studied in this regard, change in response to the glucose concentration in the medium. As a consensus, *glut1* and *glut4* mRNA levels are higher under glucose deprivation conditions than in the presence of glucose [10]. Despite extensive characterization of SGTP1 and SGTP4 from *S. mansoni* [5], no study on transcriptional regulation in response to glucose has been conducted. In addition, knowledge of the properties of SGTP2 and SGTP3 is limited.

**Fig. 1:**
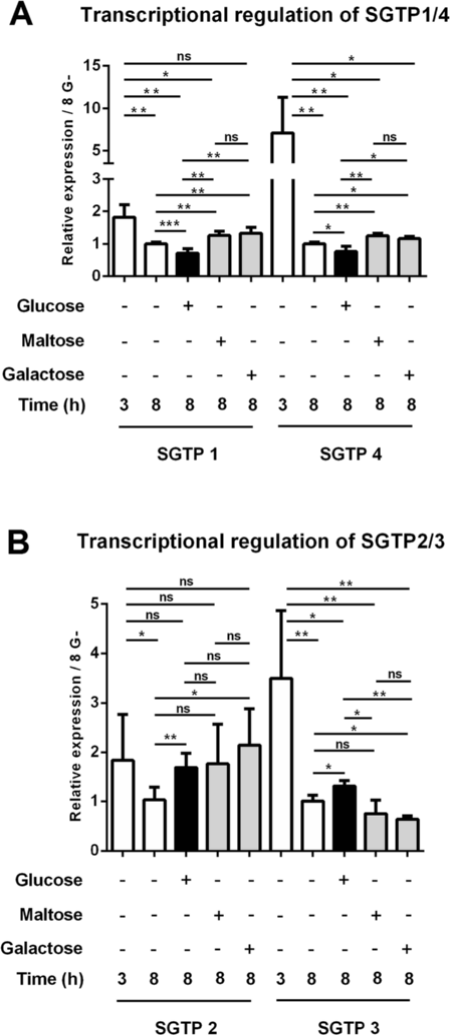
Relative expression of *sgtp1*, *sgtp2*, *sgtp3* and *sgtp4* after transformation and adding glucose. The relative expression of *sgtp1*, *sgtp4* (Panel **a**), *sgtp2* and *sgtp3* (Panel **b**) in schistosomula was determined using quantitative RT-PCR. Cercariae were mechanically transformed into schistosomula. A total of 10 000 schistosomula were incubated for 3 h with traces of glucose (0.05 mM). Subsequently, glucose, maltose and galactose were added to a final concentration of 10 mM. After 8 h of incubation, total RNA was extracted, cDNA was synthesized and quantitative RT-PCR was performed. Transcripts of *sgtp1* (*p*-*value* = 0.0006) and *sgtp4* (*p*-*value* = 0.02) were significantly downregulated after adding 10 mM glucose, while *sgtp2* (*p*-*value* = 0.002) and *sgtp3* (*p*-*value* = 0.02) were significantly upregulated after adding 10 mM glucose. Results shown are the means of three independent experiments. Gene expression data is presented relative to 8 h with 0.05 mM of glucose which was used as “calibrator sample”

Upon infection, changes in glucose concentration have been shown to be essential for *S. mansoni* [2]. Here we used an infection relevant model to evaluate the transcriptional regulation of the four glucose transporter genes from *S. mansoni* in presence of glucose. All glucose transporters were expressed in schistosomula 3 h after mechanical transformation in the presence of 0.05 mM glucose. A significant reduction in expression was observed after 8 h incubation in glucose deprivation conditions (*p*-*value* <0.01). However, when the medium was supplemented with 10 mM glucose we observed a significant change in expression pattern. While the transcriptional levels of *sgtp1* and *sgtp4* were downregulated (*p*-*value* <0.025), the transcriptional levels of *sgtp2* and *sgtp3* were upregulated (*p*-*value* <0.025) in the presence of 10 mM glucose (Fig. 1). The downregulation of *sgtp1* and *sgtp4* transcripts was specific to glucose, as the addition of galactose or maltose to the medium resulted in a significant increase (*p*-*value* <0.025) in the transcriptional levels of both genes (Fig. 1).

Our results complement those obtained by Krautz-Peterson et al. [5] since they showed that RNA interference (RNAi) of both SGTP1 and SGTP4 more strongly affected schistosomula when they were maintained in glucose-poor medium than in glucose-rich medium. In glucose deprivation conditions the parasite may increase the expression of these two glucose transporters to maximize the uptake of glucose. Thus, inhibiting the glucose transporters in glucose deprivation conditions will be more detrimental to the parasite than in the presence of glucose. The transcriptional expression levels of *sgtp2* significantly increased in the presence of galactose (*p*-*value* = 0.010), but not maltose (*p*-*value* = 0.163). In contrast, the transcriptional expression levels of *sgtp3* significantly decreased in the presence of galactose (*p*-*value* = 0.004), but not maltose (*p*-*value* = 0.125). These observations suggest that the transcriptional regulation of *sgtp2* and *sgtp3* may not be glucose-specific.

### *S. mansoni* glucose transporters cluster separately and evolve at different rates

**Fig. 2:**
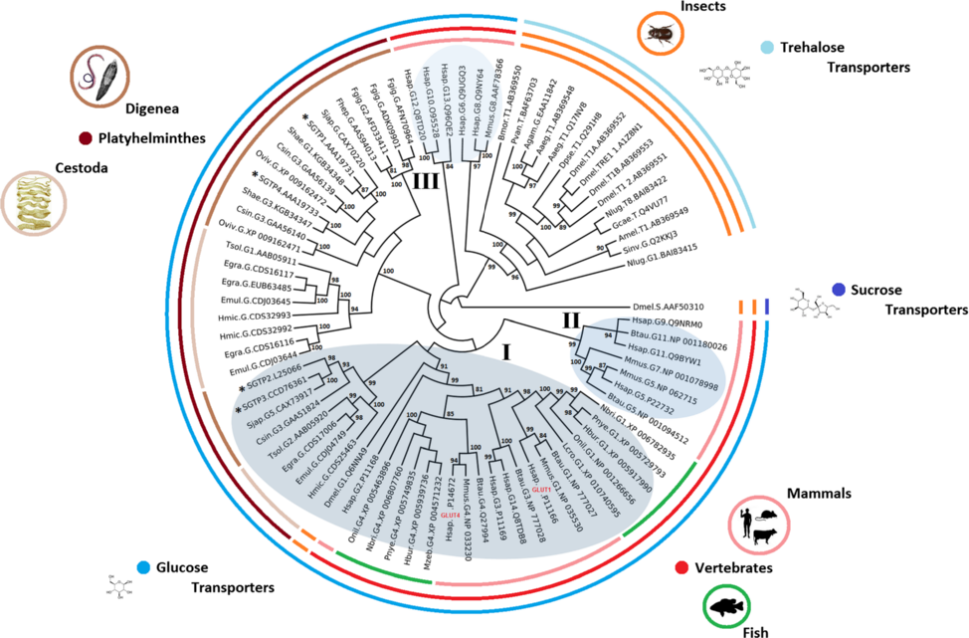
Phylogeny of the trehalose and glucose transporter family. The figure shows a phylogenetic tree of trehalose and glucose transporters from vertebrates, trematodes and insects. The topologies obtained with ML, MP and NJ methods were similar, and thus only ML is displayed. Numbers on internal branches are the bootstrap values (only >80 % are shown); the statistical support obtained with approximate likelihood ratio test (aLRT) was similar. Sequences are labeled with data collected from GenBank as name of species (four first letters), type of transporters (T: trehalose, G: glucose), number (if reported) of the sugar transporter and accession numbers. *S. mansoni* glucose transporters are indicated by asterisks. *Homo sapiens* GLUT1 and GLUT4 are highlighted in red. The classes of glucose transporters are numbered with roman numerals. *D. melanogaster* sucrose transporter was used as the outgroup. The labels for the species used are as follow: Mammals: *H. sapiens* (Hsap)*, Mus musculus* (Mmus)*, Bos Taurus* (Btau); Fish: *Oreochromis niloticus* (Onil), *Neolamprologus brichardi* (Nbri), *Haplochromis burtoni* (Hbur), *Pundamilia nyererei* (Pnye), *Larimichthys crocea* (Lcro), *Dicentrarchus labrax* (Dlab), *Maylandia zebra* (Mzeb); Insects: *Anopheles gambiae* (Agam), *Aedes aegypti* (Aaeg), *Apis mellifera* (Amel), *Bombyx mori* (Bmor), *Drosophila melanogaster* (Dmel), *Drosophila pseudoobscura pseudoobscura* (Dpse), *Gastric caeca* (Gcae), *Solenopsis Invicta* (Sinv); Cestodes: *Taenia solium* (Tsol), *Echinococcus granulosus* (Egra), *Echinococcus multilocularis* (Emul), *Hymenolepis microstoma* (Hmic), *Fasciola gigantica* (Fgig), *Fasciola hepatica* (Fhep); Flukes: *Clonorchis sinensis* (Csin), *S. haematobium* (Shae), *Opisthorchis viverrini* (Oviv), *S. japonicum* (Sjap)

Maximum likelihood (ML), maximum parsimony (MP) and neighbor joining (NJ) analyses were performed to phylogenetically characterize the *S. mansoni* glucose transporters SGTP1, SGTP2, SGTP3 and SGTP4. Amino acid sequences from glucose and trehalose transporters from the taxonomic classes Insecta, Mammalia, Teleostei, Cestoda and Trematoda were included in the phylogenetic analyses. All phylogenetic trees had the same topology with each of the above methods. Therefore, only one topology (ML) is displayed (Fig. 2).

Three classes of glucose transporters have been described in humans that form different phylogenetic clusters [9]. We found that *S. mansoni* SGTP2 and SGTP3 clustered together with vertebrate and *D. melanogaster* class I glucose transporters (Fig. 2, roman numeral I), while SGTP1 and SGTP4 formed a Platyhelminthes-specific glucose transporter cluster. The phylogeny presented in Fig. 2 is consistent with the monophyly of both Cestoda and Digenea [16] since the two clusters containing glucose transporters from Platyhelminthes, cestodes and trematodes clustered separately (Fig. 2). The phylogenetic analyses allowed us to classify SGTP2 and SGTP3 together with some glucose transporters from the flukes *S. japonicum*, *Clonorchis sinensis* and the cestodes *Taenia solium*, *Echinococcus granulosus*, *E. multilocularis* and *Hymenolepis microstoma* as class I glucose transporters. In particular, we found homologs of SGTP1, SGTP2, SGTP3 and SGTP4 in both *S. japonicum* and *S. haematobium* (Additional file 1).

**Fig. 3:**
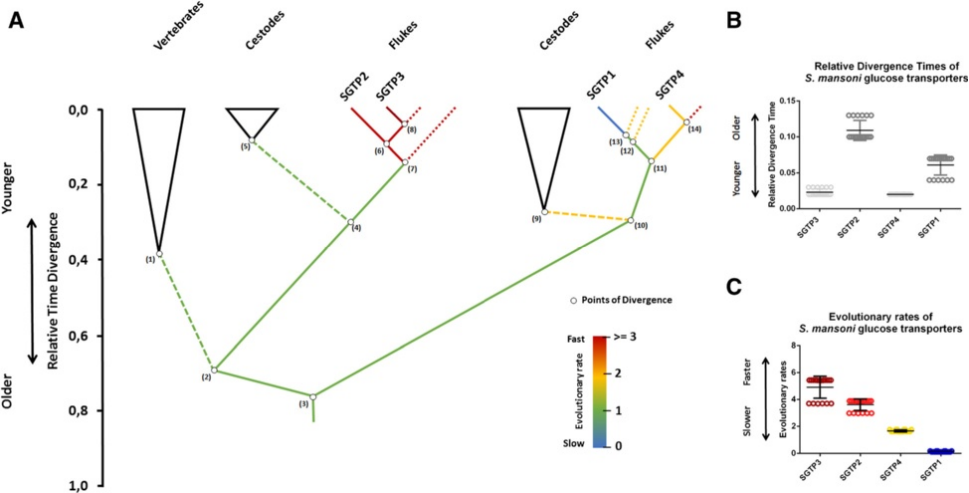
Relative divergence times and evolutionary rates of *S. mansoni* glucose transporters. The figure displays the relative divergence times of selected nodes from the ML tree topology in Fig. 1. Class I glucose transporters (node 2) and Platyhelminthes-specific cluster of glucose transporters (node 10) (Panel **a**). Divergence times for selected branching points were calculated in the 100 most parsimonious trees using the ML method based on the LG model (see Methods). Node 2 (average of relative time of divergence: 0.68, Std: 0.02) is older than node 10 (average of relative time of divergence: 0.28, Std: 0.009) with *p*-value < 0.0001. The colored scale shows the relative evolutionary rates among selected branches (Panels **a** and **b**). The evolutionary rate (evol rate) measured as the relative number of amino acid substitutions per site was calculated for selected nodes in the 100 most parsimonious trees using RelTime method [22]. The glucose transporters SGTP2 (average of evol rate: 3.6, Std: 0.42) and SGTP3 (average of evol rate: 4.9, Std: 0.81) evolved faster than SGTP1 (average of evol rate: 0.13, Std: 0.028) and SGTP4 (average of evol rate: 1.6, Std: 0.067) with a *p*-value < 0.0001 (Panel **c**)

Mammalia, Platyhelminthes and Insecta shared a common ancestor circa 695 MYA [17]. The fact that class I glucose transporters included sequences from Platyhelminthes, insects and mammals suggested that this cluster is ancestral to the Platyhelminthes-specific cluster. To test this hypothesis we determined the relative divergence times for selected nodes in the 100 most parsimonious phylogenetic trees (see Methods). Indeed, the results from this analysis showed that the cluster containing class I glucose transporters is older than the Platyhelminthes-specific cluster (Fig. 3a, node 2 vs. node 10) with *p*-*value* <0.0001. In agreement with this, SGTP2 seems to be the oldest glucose transporter in *S. mansoni* (Fig. 3b, *p*-*value* <0.0001). The relative divergence time of SGTP3 and SGTP4 suggested that these phylogenetic events coincide in time, and they are recently evolved glucose transporters compared to SGTP2 and SGTP1. The phylogenetic position of SGTP3 and SGTP4 (Fig. 2), and the relative divergence times (Fig. 3b) of these genes support the idea that they originated from SGTP2 and SGTP1 respectively, possibly due to gene duplication.

Gene duplications were reported in glucose transporters from *Homo sapiens* and *Saccharomyces cerevisiae* [18, 19]. After gene duplication, relaxation of selective constraints on duplicated genes has been observed, resulting in copies that evolve faster than their original orthologs [20, 21]. To test variations in evolutionary rates, we estimated these along branches of the glucose transporters phylogeny using the RelTime method [22]. Figure 3c shows that SGTP2 and SGTP3 evolved faster than SGTP1 and SGTP4 (*p*-*value* = 0.0001). In addition, SGTP3 and SGTP4 were found to evolve faster than SGTP2 and SGTP1, respectively with *p*-*value* <0.0001.

We further compared the evolutionary rates of SGTP3/SGTP2 and SGTP4/SGTP1 using Tajima’s relative rate test [23]. The *χ*2 test statistic was 6.50 (*p*-*value* = 0.01079) for SGTP4/SGTP1 and 0 (*p*-*value* = 1.0000) for SGTP3/SGTP2. Thus, the null hypothesis of equal rates was rejected for SGTP4/SGTP1, but not for SGTP3/SGTP2. This suggests that Tajima’s relative rate test may not be suited for distinguishing different rates of evolution in fast-evolving sequences like SGTP3/SGTP2 or simply that after divergence SGTP3 and SGTP2 evolved at the same rates. In agreement with the latter, other sequences from *S. japonicum* and *C. sinensis,* in the cluster where SGTP2 and SGTP3 are located, are also fast-evolving sequences (Fig. 3). Interestingly, the transcriptional regulation of SGTP1 and SGTP4 compared with SGTP2 and SGTP3 in the presence of glucose (Fig. 1) is in agreement with these phylogenetic and evolutionary observations.

### The rapidly-evolving glucose transporters SGTP2 and SGTP3 are encoded by intron-rich genes

The cDNA sequence encoding SGTP2 originally described by Skelly et al. [4] was constructed from a cloned sequence by the insertion of a single nucleotide in order to restore the open reading frame. The authors speculated that *sgtp2* might therefore be an expressed pseudogene and not encoded as a functional protein. Comparison of the *sgtp2* (L25066) cDNA sequence to the genome sequence allowed the reconstruction of a “genomic” predicted cDNA sequence. Three base changes were found within the encoding region, one leading to an amino acid change (Asn88Lys). Since the cDNA sequence encodes a full-length protein reconstructed by assembling the exons of the genomic sequence, we speculated that the missing nucleotide in the cDNA clone obtained by Skelly et al. [4] might represent a cloning artifact. We therefore amplified the full-length coding sequence of SGTP2 from cDNA derived from adult worms and miracidia and sequenced the clones obtained. However, the examination of twenty such sequences failed to identify a single clone corresponding to the predicted genomic sequence (not shown). A number of these contain unspliced intronic sequences, whilst others contained short indels. None of the clones encoded a full-length SGTP2 protein sequence. We are therefore unable to verify our speculation concerning the status of the *sgtp2* gene as a pseudogene or a viable coding gene. One possibility is that the functional transcript might only be produced at specific stages during the life cycle. To compare the *sgtp*2 gene organization and the potential structure and function of the SGTP2 protein, we used the transcript and protein sequence predicted by the genome. In contrast we confirmed that the sequence of SGTP3 predicted from the genome corresponds to viable transcripts present in adult worms. The transcript sequences were found to be identical to the genome prediction, but have a small deletion at the N-terminal end and to encode a further 86 amino acids at the C-terminal end. However, these sequence modifications have no effect on the core glucose transporter structure. The transcript sequence was submitted to GenBank [GenBank: KT895372].

**Fig. 4:**
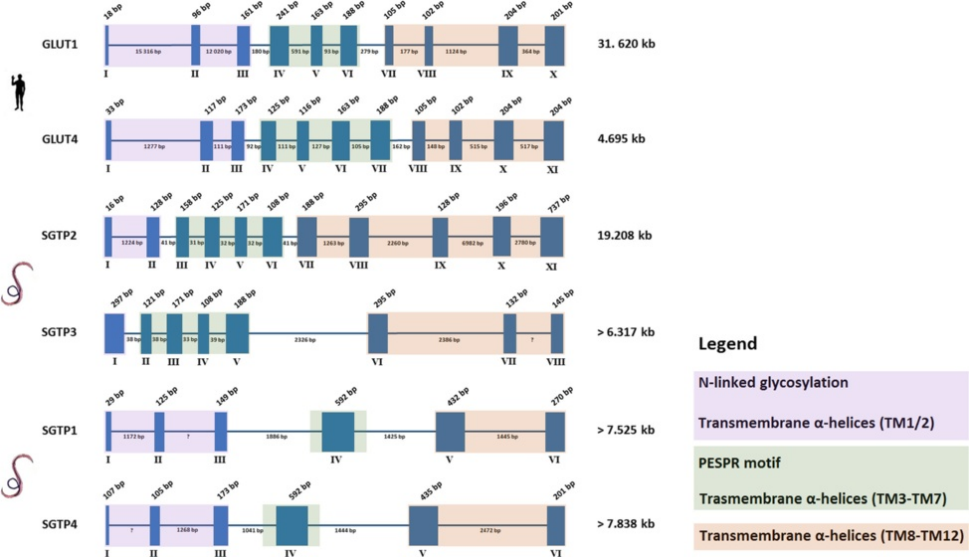
Genomic organization of *S. mansoni* glucose transporters. The genomic organization of the coding regions of *S. mansoni* glucose transporters is presented. The genomic organizations of human glucose transporters 1 (GLUT1) and 4 (GLUT4) are displayed for comparative purposes. Exons are represented by solid blue boxes and introns are represented by connecting lines. The sizes of exons and introns are shown in base pairs above and below the *boxes* and *lines*, respectively. Exons are numbered with roman numerals beneath the *boxes*. Exons coding for different regions of the proteins are *highlighted in purple, green and orange*. Interrogation marks are showing intron positions were information is missing from genome sequences (see Table 1 for complementary information)

The schistosome genome sequence and the derived exon/intron structure shown in Fig. 4 and Table 1 are aligned with published transcript sequences of SGTP1 and SGTP4, the transcript sequence of SGTP2 predicted from the genome, and the predicted transcript sequence of SGTP3. We analyzed the gene structure of human *glut1*, and collected that of *glut4* from the literature [24], for comparison, since our phylogenetic analyses placed the human GLUT1 and GLUT4 within the same clade as SGTP2 and SGTP3. The analysis shows that the gene structures of *sgtp1* and *sgtp4* are similar to each other, but different from those of *sgtp2* and *sgtp3*, which are also similar to each other. Both *sgtp1* and *sgtp4* have 5 introns, four of which are large (>1 000 bp), the size of the fifth was not evaluated due to missing sequence data. In contrast, *sgtp2* has 10 introns and *sgtp3* has 7 introns. Apart from the first intron of *sgtp2*, which is large and not conserved in *sgtp3*, the first introns of these two genes are small, between 31 bp and 41 bp, whereas the introns at the 3’ end of both genes are large. Moreover, the sizes of exons 4 to 9 of *sgtp2* and 2 to 7 of *sgtp3* are identical and the positions of these introns with respect to the encoded amino acid sequence of the corresponding proteins are conserved. Comparison of *sgtp1*, *sgtp4*, *sgtp2*, *sgtp3* and *glut1* genes (Table 1) shows that 3 intron positions are perfectly conserved between all, and that the position of introns 1 and 2 is conserved between all but *sgtp3*. In addition, *glut1* shares an intron position (intron 5) with *sgtp2* (intron 6) and *sgtp 3* (intron 4) that is not present in *sgtp1* or *sgtp4*. Overall, this analysis underlines the close relationship between *sgtp2* and *sgtp3*, and *sgtp1* and *sgtp4* that correlates well with our phylogenetic analysis.

**Table 1:**
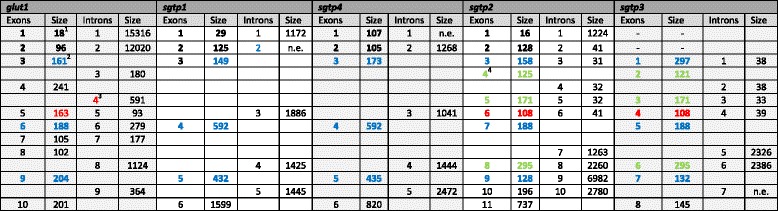
Genomic structure and organization of *S. mansoni* glucose transporters

Exon and intron sizes are in base pairs

In bold: Exons for which the exon-intron junction at the 3’ end is conserved between different glucose transporter genes

In blue: Exons for which the exon-intron junction at the 3’ end is conserved among all selected glucose transporter genes

In red: Exons for which the exon-intron junction at the 3’ end is conserved only between human *glut1*, *sgtp2* and *sgtp3*

In green: Exons for which the exon–intron junction at the 3’ end is conserved only between *sgtp2* and *sgtp3*

*n.e.* not evaluated (information missing from genome sequences)

### *S. mansoni* glucose transporters show typical molecular signatures

**Fig. 5:**
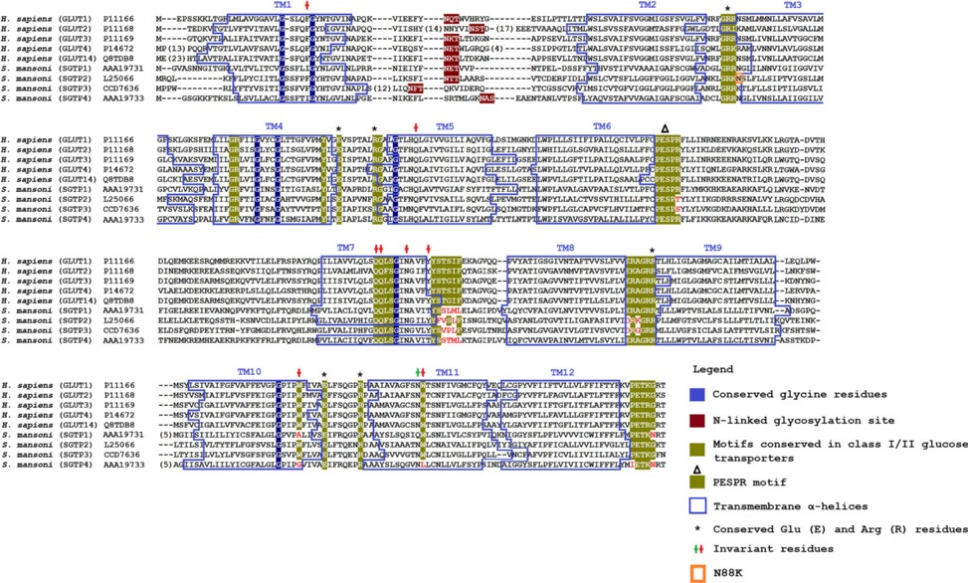
Alignment of members of glucose transporters class I. Class I glucose transporters amino acid sequences from human (GLUT1-4 and GLUT-14) and *S. mansoni* (SGTP1, SGTP2, SGTP3 and SGTP4) were aligned using MAFFT. Relevant motifs among human class I/II glucose transporters and *S. mansoni* are *highlighted*. Annotation of relevant motifs was done using previous reports [21, 25, 26]. The 12 transmembrane (TM) topologies, predicted using TMHMM Server v. 2.0 [27], are indicated. The positions of the invariant residues of XylE (*green*) and GLUT1 (*red*) are marked. The substitution of Asn(N)88 by Lys(K)88 found in the genome for SGTP2 is *boxed* (*orange*). The number of amino acid insertions that resulted from the alignment are shown in *brackets*

Despite SGTP1 and SGTP4 being clustered separately from class I and II glucose transporters, they share several molecular properties with these classes (Fig. 5). Human class I glucose transporters comprise GLUT1, GLUT2, GLUT3, GLUT4 and GLUT14 with GLUT1 being the first isoform cloned described by Mueckler et al. [28]. All members of GLUT family isolated from humans possess 12 transmembrane (TM) α-helices [9], structural patterns that are also present in some glucose transporters from Insecta [29]. Using the TMHMM server we predicted the presence of the 12 TM α-helices in SGTP1-4 and compared their positions with human class I glucose transporters (Fig. 5). The positions of these TMs were conserved in the four glucose transporters from *S. mansoni*, except for the lack of TM7 in SGTP3. The only remarkable difference between the amino acid sequence of SGTP3 and the other glucose transporters at TM7 is the presence of two phenylalanine residues (Fig. 5). However, the modelling analysis (see below) showed that SGTP3 has the typical 12 TM α-helices. The discrepancy between TMHMM and modelling predictions regarding TMs in SGTP3 can only be solved through the experimental determination of the structure of this transporter. Probably due to differences in the algorithm used, the positions of our predicted TM differ slightly from those reported by Skelly et al. [4] for SGTP1 and SGTP4. We then compared the sugar transporter signatures [9, 30] of human and *S. mansoni* glucose transporters. Some glycine (Gly) residues critical for structure stabilization in the GLUT family [28] were also conserved in the TM α-helices 1, 4, 5, 7 and 10 of *S. mansoni* glucose transporters (Fig. 5). The glutamic acid (Glu) and arginine (Arg) residues implicated in conformational alteration [31] and membrane topology [32] in human GLUTs were also present in cytoplasmic loops 2, 4, 8, and 10 of *S. mansoni* glucose transporters (Fig. 5).

Some exclusive characteristics of class I/II glucose transporters in humans are conserved in *S. mansoni*. For example, the N-linked glycosylation site (Fig. 5) in the first extracellular loop between TM1 and TM2 [33] and the motif PESPR in the sixth intracellular loop between TM6 and TM7 (Fig. 5). SGTP2 and SGTP3 showed an amino acid change in the last Arg of the motif PESPR to threonine (Thr) and serine (Ser), respectively. Despite the improbability of deducing substrate specificity or transport kinetics from the primary amino acid structure of class I glucose transporters, GLUT1, 3 and 4 are known to transport glucose, and not fructose, via a QLS sequence present in TM 7 [34]. This triad sequence has been implicated in substrate binding and is conserved in *S. mansoni* SGTP1, SGTP4 and SGTP3 (Fig. 5), but not in SGTP2. In SGTP2 there is a substitution of leucine (Leu) by phenylalanine (Phe) and the same substitution was found in the human GLUT2. Human GLUT2 has low affinity for glucose (*K*_*m*_ = 17 mM), which is uncommon among the known members of the GLUT family [35], but has a high affinity for glucosamine (*K*_*m*_ ≈ 0.8 mM) [36]. *S. mansoni* SGTP2 does not seem to transport glucose in *Xenopus* oocytes at 10 mM glucose [4], however, further studies should clarify whether this molecule shows a higher *K*_*m*_ for glucose or whether it has an affinity for glucosamine as does GLUT2. The presence of glucose transporters with different affinities for glucose is extremely important for the regulation of glucose uptake. For instance, under high glucose concentration conditions an increase in intracellular glucose reduces the glucose influx 50 % in yeast cells expressing high-affinity glucose transporters [37]. Thus, by regulating the expression profile of the different glucose transporters with different kinetic properties, yeasts avoid affecting their glucose uptake, which is directly dependent on extracellular glucose concentration [38]. Despite the fact that the hypothesis of glucose transporters with different affinities for glucose in *S. mansoni* is appealing and matches the expression profile in Fig. 1, our biophysical analysis (below) suggests that neither SGTP2 nor SGTP3 transport glucose.

### The predicted tertiary structural conformations of *S. mansoni* glucose transporters are homologous to GLUT1 and XylE

Upon substrate binding, the tertiary structure of most proteins undergoes conformational changes that coordinate their function. By comparing the structural differences of XylE and GLUT1, Deng et al. [12] were able to hypothesize on the conformational changes of glucose transporters during glucose binding and diffusion. This working model of glucose diffusion predicts that transporters prefer an outward (towards the extracellular), open, glucose-free conformation (i.e., the initial conformation). Glucose association (i.e., conformation of XylE) and disassociation (i.e., conformation of GLUT1) trigger the migration of glucose towards an environment with a lower concentration – i.e., the intracellular compartment [12]. The final conformation, glucose-free occluded (by the intracellular α-helices), remains to be structurally resolved.

**Fig. 6:**
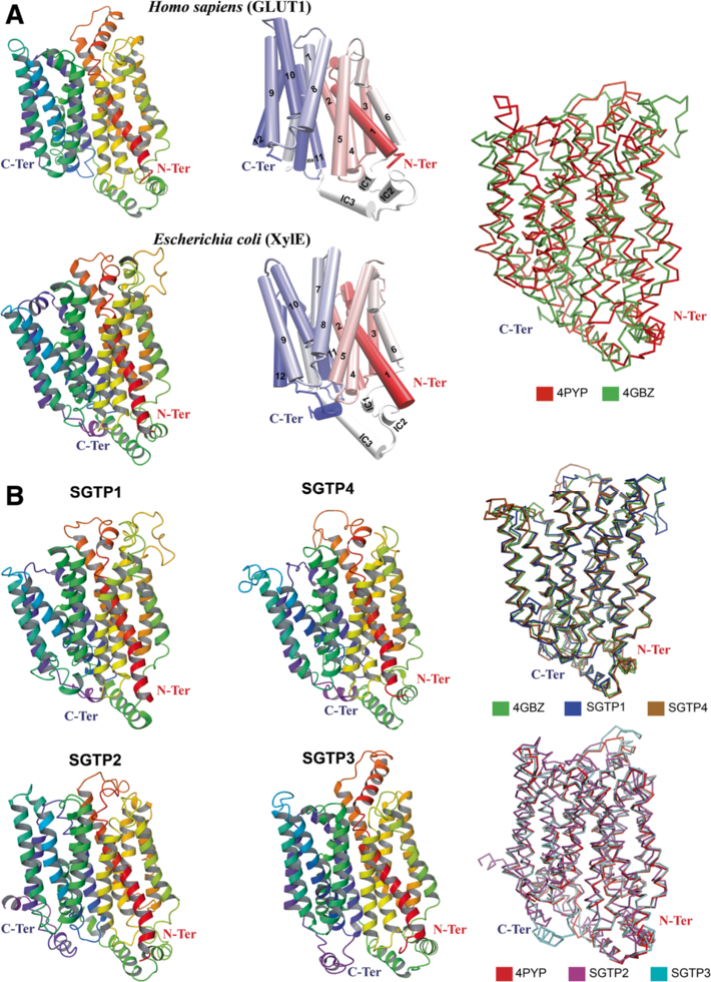
XylE and GLUT1 compared to the tertiary predicted structures of *S. mansoni* glucose transporters. Panel **a** (from left to right) shows the crystal structures of glucose transporters GLUT1 of *H. sapiens* [PDB: 4PYP] and XylE of *E. coli* [PDB: 4GBZ]. The transmembrane (TM) α-helices (*middle*) are numbered (1–12) accordingly (IC = intracellular) with the α-carbon backbone structural alignment of both crystal structures (GLUT1 = red and XylE = green; far right). Panel **b** shows the tertiary predicted structures of *S. mansoni* glucose transporters (left) with the structural alignment (*right*) of SGTP1 (blue) and SGTP4 (*brown*) with XylE of *E. coli* (*green*), and SGTP2 (*magenta*) and SGTP3 (*cyan*) with GLUT1 of *H. sapiens* (*red*). Structures are color-coded from the amino-terminus (N-ter; *red*) to the carboxyl-terminus (C-ter; *blue*). The structural alignment of the α-carbon backbone was performed using the default tool of Schrodinger’s Maestro program

As proof of principle for our simulations, we submitted the modelled *S. mansoni* glucose transporters to the DALI server [39] to find homologous crystal structures with the best matching conformation. The crystal structures GLUT1 of *H. sapiens* [PDB: 4PYP] and XylE of *E. coli* [PDB: 4GBZ] were among the top candidates. This provided two opportunities to control our simulations since, (i) glucose is not the substrate of XylE, it is a competitive inhibitor and (ii) XylE and GLUT1 are the two sequential intermediate structural conformations during glucose binding and diffusion, respectively [12]. The conformational changes during binding occur mainly in TM α-helices 1, 4, 7 and 10 that cause the occlusion by the intracellular α-helices to open, thereby permitting diffusion [12]. Root mean square deviation (RMSD) is frequently used in structural bioinformatics to measure the average distances between atoms from two or more protein structures. The superposition in Fig. 6a graphically shows these overall structural differences – the RMSD between XylE [PDB: 4GBZ] and GLUT1 [PDB: 4PYP] is 3.2 Å.

Figure 6b shows the predicted tertiary structures of *S. mansoni* glucose transporters SGTP1-SGTP4 and their structural similarity to GLUT1 and XylE. Both SGTP1 (RMSD 1.0 Å) and SGTP4 (RMSD 1.3 Å) are structurally homologous to XylE. SGTP2 (RMSD 2.3 Å) and SGTP3 (RMSD 0.6 Å) are structurally homologous to GLUT1. The higher RMSD for SGTP2 is mainly caused by differences in TM α-helices 9 and 10, intracellular α-helices and the C-terminus. These opposing conformations allow us to examine the stability of glucose within the active site of SGTP1 and SGTP4 and the inability of glucose migration (or diffusion) by SGTP2 and SGTP3.

### Residue dynamics reveal how glucose affects invariant residues of glucose transporters involved in binding

The dynamics of key residues during substrate binding vary depending on the substrate and the protein. Since 1958, the key-lock theory to explain protein-substrate complexes has been revised by Koshland [40] who proposed an induced-fit process. The induced-fit theory demonstrates that during protein-substrate interactions, conformational changes occur between both the protein and substrate to accommodate the complex. These molecular interactions subsequently cause the global conformational changes aforementioned in Fig. 6. To understand how glucose will interact with key residues of glucose transporters, we performed an induced-fit docking simulation using the PELE server (see Methods).

**Fig. 7:**
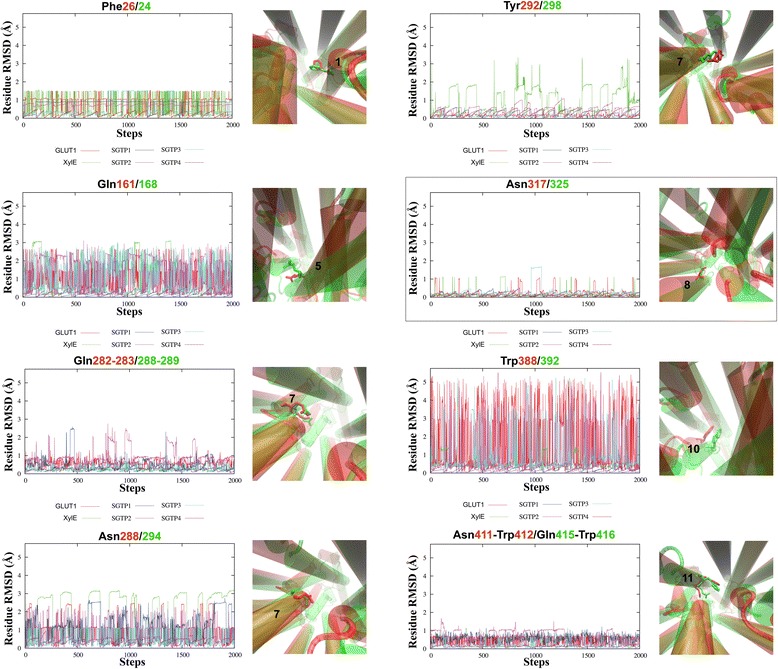
Protein side chain dynamics during glucose induced-fit docking of XylE, GLUT1 and *S. mansoni* glucose transporters. The *line graphs* depict the conformational changes of protein side chains in proximity to glucose for glucose transporters GLUT1 (*red*), XylE (*green*), SGTP1 (*blue*), SGTP2 (*magenta*), SGTP3 (*cyan*) and SGTP4 (*brown*). The accepted PELE steps (*x-axis*) for all simulations were used to calculate the root mean square deviation (RMSD; given in Å) of side chain dynamics (*y-axis*) – see Methods. Next to each line graph are the respective initial positions for each residue (sticks) of GLUT1 (*red*) and XylE (*green*) crystal structures with the number indicating the parent TM α-helix (tubes). The title of each line graph is color-coded respectively to GLUT1 and XylE. The *boxed*-in panel is the negative control since it does not directly affect in substrate binding [13]. (Note: The graphical structural representations are a bird’s-eye view of GLUT1 and XylE.)

Several conserved, invariant residues among glucose transporters (namely, GLUT1- GLUT4) were reported for XylE that, when mutated to alanine (Ala), reduce the affinity of substrate binding (highlighted in Fig. 5). These XylE invariant residues are responsible for the competitive inhibitory properties of glucose [13] and showed altered conformations between XylE and GLUT1 crystal structures. The differences in conformation between XylE (green) and GLUT1 (red) invariant residues are clearly depicted in the structural representations of Fig. 7. Large residue conformational shifts between XylE and GLUT1 are seen in Gln161/168 (TM5), Tyr292/298 (TM7) and Trp388/392 (TM10). Both TM7 and TM10 are involved in the two sequential intermediate structural conformations during glucose binding [12]. These homologous residues for SGTP1 and SGTP4 are, however, mutated – in TM7 as Tyr292/298Thr (SGTP1 and SGTP4), and in TM10 as Trp388/392Ala (SGTP1) and Trp388/392Gly (SGTP4) (Fig. 5). To simplify matters we refer to the invariant residue positions in the order GLUT1/XylE, hereafter.

The residue dynamics depicted in the line graphs of Fig. 7 show that Gln161/168, Gln282-283/288-289, Asn288/294, Tyr292/298 and Trp388/392 undergo large conformational changes during induced-fit docking of glucose (compared with the dynamics of the negative control, Asn317/325 – see Fig. 7 legend). Fig. 7, however, shows that these dynamics differ depending on the glucose transporter. XylE and GLUT1 show high perturbations for Gln161/168 and Asn288/294, but have opposing dynamics for Tyr292/298 (XylE) and Trp388/392 (GLUT1). The *S. mansoni* glucose transporter SGTP4 showed extremely low perturbations for all binding residues, perhaps due to its lower affinity for glucose compared with SGTP1 [4]. In contrast, SGTP2 possesses the most invariant residues with high perturbations, namely for Gln161/168, Gln282-283/288-289, Asn288/294 and Trp388/392. Both SGTP1 (Gln282-283/288-289 and Asn288/294) and SGTP3 (Gln161/168 and Trp388/392) only showed high perturbations for two invariant residues (Fig. 7). There is a kink in TM7 that contributes to the coordination of the substrate in the active site of glucose transporters in the proximity of polar residues Gln282-283/288-289 and Asn288/294 and the aromatic residue, Tyr292/298 [13]. The benzene ring of Tyr292/298 may act as “trapdoor” switch for glucose since it is thought to maintain the open-inward conformation of GLUT1 by interacting with TM4 [12].

### Biophysical properties of *S. mansoni* glucose transporters provide insights into glucose affinity

The induced-fit simulations revealed how invariant residues behave upon binding (since the algorithm constrains glucose migration within the active site), but what would happen if glucose were unconstrained? To answer this question, we simulated an unconstrained glucose migration starting from the active site. Due the importance of Tyr292/298 in maintaining the open-inward conformation of glucose transporters [12], we used it to triangulate glucose migration from its initial position in the active site. Additional file 2 shows distinct glucose migration patterns for GLUT1 and XylE. In accordance with the structural similarities (Fig. 6), transcript expression (Fig. 1) and evolution (Figs. 2 and 3), *S. mansoni* glucose transporters group together in glucose migration patterns showing that in SGTP2 and SGTP3 glucose migrates similarly to GLUT1, while SGTP1 and SGTP4 are similar to XylE. These migration patterns seem to show glucose either slowly diffusing away from the active site (i.e., XylE and SGTP4) or being ejected from it (i.e., GLUT1, SGTP2 and SGTP3). In SGTP1, however, glucose seems to stay within 10 Å of the active site (Additional file 2). This concentrated positioning within the active site agrees with the higher affinity that SGTP1 has for glucose compared with other SGTPs [4].

**Fig. 8:**
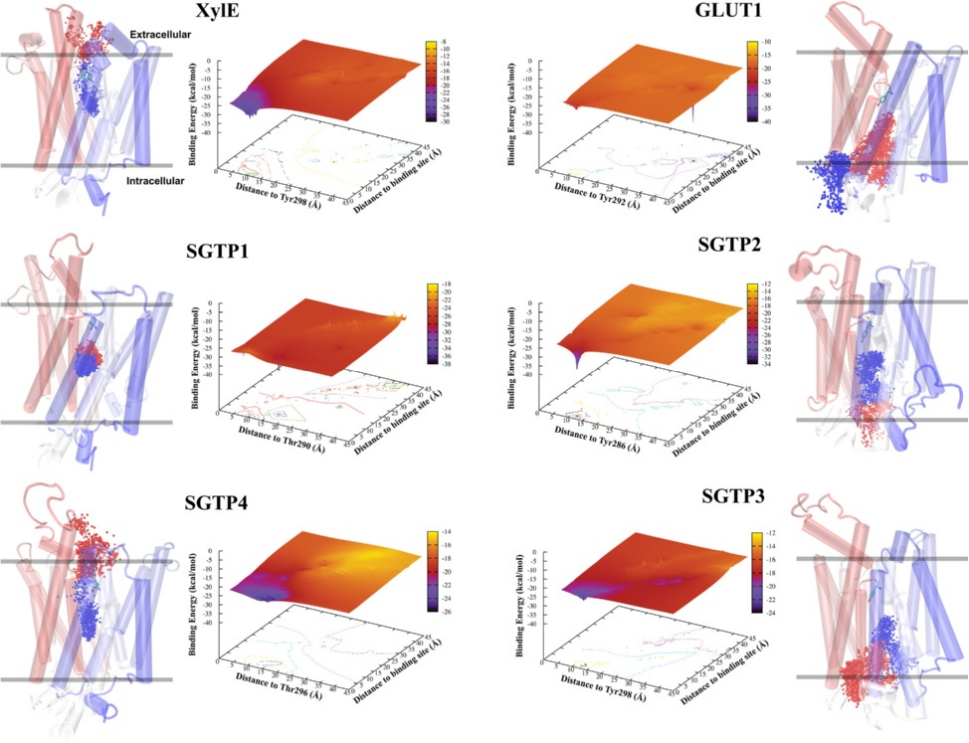
Biophysics of glucose migration from the active sites of XylE, GLUT1 and *S. mansoni* glucose transporters. All 3D plots show the mapping of glucose binding energy (kcal/mol; z-axis) compared to the triangulation of the glucose migratory distance (given in Å) from the Tyr292/298 homologous residue (*x-axis*) and the active site (*y-axis*) for GLUT1, XylE, SGTP1, SGTP2, SGTP3 and SGTP4. The spectrum indicates the energy (kcal/mol) from a favorable glucose binding state (decreasing value; *violet-blue*) to an unfavorable binding state (increasing value; *red-yellow*). The contour base on the Cartesian coordinates is a 2D representation of the topology for the binding energy map. Next to each 3D plot are the graphical structural representations from the cluster analysis (see Methods) depicting glucose migration from favorable binding energies (*blue*) to unfavorable ones (*red*). One oxygen atom (O5) was used to represent each glucose migration step away from the active site. The final position of Tyr292/298 is shown for each structure and each are color-coded from the N-ter (*red*) to the C-ter (*blue*). The *lines* indicating the position of the membrane is an approximation based on the position in Fig. 5 – only for demonstration purposes

In order to determine the biophysics of glucose affinity and the direction of migration patterns (towards the extracellular or intracellular compartments) we performed two types of analyses: (i) a cluster analysis to visualize the direction, and, (ii) an incorporation of 3D energy mapping of glucose migration to localize energy favorable clusters. Figure 8 shows that XylE and GLUT1 possess conversely energy favorable glucose migration patterns. This is strictly caused by the structural conformation of these transporters since both are occluded by the extracellular TMs, but differ in the conformation of intracellular α-helices (closed for XylE and open for GLUT1). The open-inward conformation of GLUT1 is the characteristic that permits the diffusion of glucose within the cell. The distinct migration patterns and energy signatures of *S. mansoni* glucose transporters, however, may explain their individual response to glucose (Fig. 8).

Although SGTP2 and SGTP3 are structurally similar to GLUT1, the energy signatures for both are not comparable to GLUT1. The energy signature difference between SGTP2 and SGTP3 may be due to their slight structural deviations (as mentioned above and shown in Fig. 6). Functional studies showed that SGTP2 does not transport glucose, even at 10 mM concentration [4]. Our transcriptional analysis shows that SGTP2 and SGTP3 have opposing responses to glucose compared with SGTP1 and SGTP4 (Fig. 1), and phylogenetically group together (Fig. 2). These functional and evolutionary insights may explain glucose migration for SGTP2 and SGTP3 (Fig. 8), since it migrates from an energy favorable state (i.e., the active site) to an unfavorable energy state (i.e., towards the intracellular compartment). Our results showed that transcriptional regulation of SGTP2 and SGTP3 respond differently to maltose and galactose than do SGTP1 and SGTP4 (Fig. 1). All these results suggest that glucose is not the native substrate for SGTP2 and SGTP3, but may act as an inhibitor (as in XylE) since both show energy favorable states within the active site (Fig. 8).

Glucose affinity is higher for SGTP1 (*K*_*m*_ = 1.3 mM) than for SGTP4 (*K*_*m*_ = 2 mM) [4] and this affinity is clearly demonstrated in Fig. 8. For SGTP4 glucose migrates from an energy favorable state (i.e., the active site) to an unfavorable energy state (i.e., towards the extracellular compartment). In contrast, glucose spends the majority of the simulation within the active site of SGTP1 with unfavorable energy states. We do see, however, that a smaller glucose cluster migrates towards the intracellular compartment with energy favorable states (Fig. 8). This may be due to the similarity in structural conformation between SGTP1 and XylE (with closed intracellular α-helices), thereby impeding its diffusion by trapping glucose within the active site. The fact that SGTP1 differs from XylE and SGTP4 in energy and migration patterns may be due to the fact that SGTP1 possesses a higher affinity for glucose [4] and that xylose, not glucose, is the native substrate for XylE [13].

## Conclusions

SGTP2/3, not SGTP1/4, are relatively close to human GLUT1/4. This conclusion is based on our observations. The phylogenetic analysis shows that SGTP1/4 belong to a Platyhelminthes-specific glucose transporter class, while SGTP2/3 clearly belong to class I glucose transporters together with vertebrates and insect glucose transporters. Both SGTP2 and SGTP3 show a genome organization similar to that of GLUT1/4 and different to SGTP1/4. SGTP2 seems to be the ancestral glucose transporter in *S. mansoni* and GLUT1 is a structural homolog of SGTP2/3. Thus, the capacity of SGTP1/4 to transport glucose may have evolved independently, while SGTP2/3 apparently lost this capacity after the divergence from the last common ancestor with the other glucose transporters of class I. Overall, these observations may permit the development of specific inhibitors for *S. mansoni* glucose transporters.

A recent review [8] suggested that blocking glucose uptake, as the first step of glucose metabolism, represents an appealing strategy for developing drugs to produce starvation, energy supply deficiency, and mortality in parasitic worms. This is particularly necessary since praziquantel is currently the only treatment available for schistosomiasis and its massive use, notably in sub-Saharan Africa, has led to concerns about the potential for the development of resistance [41]. Glucosides, plant-derived compounds with glucose bound to a functional, non-sugar group (aglycone), have been shown to inhibit glucose diffusion in *S. mansoni* [4, 42] and could therefore represent lead compounds for developing drugs targeting glucose transport. With the advent of computational biology, the ability to rationally design drugs for specific protein targets has greatly advanced. For example, using the *S. mansoni* glucose transporter structures, the aglycone could be altered to computationally test for stability and affinity of the designed glucoside(s). The top glucoside candidate(s) could then be experimentally analyzed for effectiveness. Since glucosides are non-transportable [43] they could “trap” themselves in the active site to maintain the structural conformation similar to SGTP1 in Fig. 8 – i.e., the glucose-bound, occluded conformation of XylE. One of the caveats of the PELE algorithm is that it cannot perform large protein conformational changes, as those described by Deng et al. [12]. Such simulations to rationally design drugs will benefit from other molecular dynamics software such as Desmond [44].

## Methods

### Ethics statement

All animal experimentation was conducted in accordance with the European Convention for the Protection of Vertebrate Animals used for Experimental and other Scientific Purposes (ETS No 123, revised Appendix A) and was approved by the committee for ethics in animal experimentation of the Nord-Pas de Calais region (Authorization No. AF/2009) and the Pasteur Institute of Lille (Agreement No. A59-35009).

### Parasite material

A Puerto Rican strain of *S. mansoni* was maintained in the laboratory using the snail, *Biomphalaria glabrata* as its intermediate host and the golden hamster, *Mesocricetus auratus* as a definitive host. Schistosomula were obtained by mechanical transformation from cercariae released from infected snails as previously reported [45]. Schistosomula were washed three times in serum and glucose-free DMEM medium (Life sciences, Carlsbad, USA) supplemented with traces of glucose (0.05 mM) then incubated at 37 °C, in an atmosphere of 5 % CO_2_. After three hours of incubation, glucose, maltose or galactose were added to the medium at a concentration of 10 mM each, and the schistosomula were incubated for another eight hours. A total of 10 000 parasites were used per condition and experiments were carried out in triplicate.

### RNA isolation and quantitative RT-PCR

Total RNA was isolated from the different stages of *S. mansoni* with TRIzol reagent (Invitrogen, Carlsbad, USA) according to the manufacturer’s instructions. Complementary DNAs (cDNA) were obtained by reverse transcription of total RNA using the Thermoscript RT-PCR System (Invitrogen, Carlsbad, USA). The cDNAs were then used as templates in triplicate assays for RT-PCR amplification using the KAPA SYBR FAST ABI Prism kit (Kapa Biosystems, Boston, USA), and ABI PRISM 7000 sequence detection system. We used previously reported primers for *S. mansoni sgtp1* and *sgtp4* [5]. The primers for *sgtp2* and *sgtp3* were designed for this study: *sgtp2*F 5’ TTTACCTTCGAGGGCAAGAT 3’ and *sgtp2*R 5’ CACCGCAAGTATGGAATACG 3’, *sgtp3*F 5’ GCAGCAACTCTCAGGAATCA 3’ and *sgtp3*R 5’ACACAATAACCGCTCCAACC 3’. The ratios of relative expression were calculated using the 2^-ΔΔCt^ ratio [46] with *S. mansoni* α-tubulin as the endogenous control gene [47]. The statistical significance between groups was evaluated using the unpaired non-parametric Mann Whitney’s test in the GraphPad 6 Prism program (GraphPad Software Inc.). Differences were considered significant when *p*-*value* <0.05.

### Phylogenetic analyses

Sequences were collected from GenBank for phylogenetic analyses of sugar transporter proteins from mammals (3 species), fish (teleosts, 7 species), insects (8 species), cestodes (6 species) and flukes (4 species). A *Drosophila melanogaster* sucrose transporter was introduced as an outgroup. The species, sugar specificity and accession numbers of each sequence is provided in Fig. 2. Glucose transporter amino acid sequences were aligned using MUSCLE (v3.7) configured for highest accuracy [48] and non-aligned regions were removed with Gblocks (v 0.91b) [49]. Thus, the final alignment contained 333 gap-free amino acid positions. The best-fit model of the sequence evolution was selected based on Akaike Information Criterion (AIC), Corrected Akaike Information Criterion (cAIC) and Bayesian Information Criterion (BIC) implemented in Datamonkey [50]. The LG [51] model, which had the lowest values of AIC, cAIC and BIC, was chosen for subsequent phylogenetic analyses. Maximum likelihood (ML), maximum parsimony (MP) and neighbor joining (NJ) methods, implemented in MEGA 6 [52], were used to obtain the best tree topologies for each method. A proportion of Gamma distributed and invariants sites (G + I) were estimated in MEGA for each phylogenetic method. Reliability of internal branches was assessed using the bootstrapping method (1 000 bootstrap replicates) and the approximate likelihood ratio test (aLRT – SH-Like) implemented in MEGA and PhyML, respectively [52, 53]. Graphical representation and editing of the phylogenetic tree was performed with EvolView [54].

### Molecular clock and evolutionary rates

First, the likelihood ratio test (LRT) [55] was used to test the molecular clock hypothesis (i.e., all tips of the tree are equidistant from the root of the tree) on the aforementioned phylogenetic trees [52]. The LRT rejected the null hypothesis of equal evolutionary rate throughout the tree at a 5 % significance level (*p*-*value* = 0) for both topologies (ML and MP). Therefore, relative time divergence was determined [56]. Time trees were generated using the RelTime method [22], as implemented in MEGA 6 [52]. RelTime is a useful method to estimate relative lineage-specific evolutionary rates and relative divergence times without requiring the pre-specification of statistical distribution of lineage rates and clock calibrations [22]. To calculate evolutionary rates, RelTime assumes that the elapsed time of two sister lineages from their most recent common ancestor is equal [22]. Divergence times for all branching points were calculated using the Maximum Likelihood method based on the LG model. The MP tree(s) were used to estimate the relative divergence times of glucose transporters in different taxa with special emphasis on *S. mansoni*. To account for statistical errors in our analysis, the 100 most parsimonious trees were searched using the Min-mini heuristic algorithm [57], and the relative divergence times of the selected nodes was determined in all the trees. The Shapiro-Wilk normality test [58] rejected normal distribution of the obtained relative divergence times (*P* <0.0001), therefore a paired Wilcoxon test was used to test whether the difference between divergence times of selected nodes were significant [56]. The GraphPad 6 Prism programme (GraphPad Software Inc.) was used to perform both Shapiro-Wilk and Wilcoxon tests. Evolutionary rates (measured as the relative number of amino acid substitutions per site) were calculated using the RelTime method [22], implemented in MEGA 6 [52]. To further compare the evolutionary rates of SGTP3/SGTP2 and SGTP4/SGTP1 we used Tajima’s relative rate test [23]. This method tests whether two sequences have equal rates of evolution using a third sequence as outgroup. The equality is tested using the chi-square (*χ*2) test. When the observed *χ*2 is significantly higher (*p*-*value* <0.05) than expected, the null hypothesis of equal rates can be rejected. Thus, to perform Tajima’s relative rate test, Fhep.G.AAS94013 and Tsol.G2.AAB05920 sequences (see Fig. 2) were used as outgroups to compare the rates of SGTP3/SGTP2 and SGTP4/SGTP1, respectively.

### Analysis of genome organization and transcript variants

The cDNA sequences of the four encoded *S. mansoni* glucose transporters were compared with genomic sequences available at the Wellcome Trust Sanger Institute Blast server (http://www.sanger.ac.uk/cgi-bin/blast/submitblast/s_mansoni) and GeneDB (http://www.genedb.org/Homepage/Smansoni). Intron-exon junctions were manually detected (5’GT and 3’AG) using sequence alignments constructed with Megalign (DNAStar Inc.). For this analysis, the cDNA sequences for SGTP1 [GenBank: L25065], SGTP2 [GenBank: L25066] and SGTP4 [GenBank: L25067] published by Skelly et al. [4] were used. In order to verify and complete the predicted transcript sequences of SGTP2, we carried out 5’ and 3’ RACE (GeneRacer kit, Invitrogen, Carlsbad, USA) using the oligonucleotides: *sgtp2*F1 5’ GTAAAACACAATCAATGAGACAACTG 3’, *sgtp2*R1 5’ GTAGAAAATAACTGGATAGATGACGA 3’ and *sgtp2*R2 5’ ATGGGAAATAAAACAAAATAGAACAA 3’, based on the predicted sequence. In the case of SGTP3, a similar strategy was adopted, based on the sequence (Smp_127200) predicted by genome annotation [59]. We carried out 5’ and 3’ RACE PCR using the oligonucleotides: sgtp35.1 5’ CTGCCGCGCCACGTGACTTTATT 3’, *sgtp3*5.2, 5’ TTGTTGGGATAGAAAGAAGGAAT 3’, *smgtp3*3.1 5’ ATCTTGGGTTGGAGCGGTTATTGT 3’, smgtp33.2 5’ TCACTCAAGAATATAGGGATGC 3’. Subsequently, the full-length coding sequence was amplified using oligonucleotides: *smgtp3*FL1 5’ CACTGACATGGACTGAAGGAGT 3’, *smgtp3*FL2 5’ TGCTACGAGTTTCTGCTTCTCATGC 3’, *smgtp3*FL3 5’ TTAATGATAGTACTGCACTGATTTA 3’, smgtp3FL4 5’ AGAATCGTTTTACCGGTATGATTGT 3’. Verification and the search for variants of the full-length coding sequences were carried out by PCR using the Advantage 2 Polymerase mix according to the manufacturer’s instructions (Clontech, Mountain View, USA). PCR products were purified from agarose gels using the extraction kit Wizard SVGel and PCR clean-up system (Promega) and then inserted into pCR2.1-TOPO vector to chemically transform competent *E. coli* cells (One-Shot TOP10, Invitrogen). Eurofins Genomics GmbH sequenced the clones. Analysis and alignment of the sequences were performed using the LASERGENE package (DNAStar).

### Tertiary protein modelling

To approximate an accurate tertiary model of the *S. mansoni* glucose transporters SGTP1, SGTP2, SGTP3 and SGTP4 we used several protein structure prediction servers, namely FOLDpro [60], I-TASSER [61], 3D-Jigsaw [62], LOOPP [63], Phyre2 [64] and SwissModel [65]. To assess the quality of the output models and to choose the top candidates we used Resprox [66], Qmean [67], ModFOLD [68]. We then manually inspected the top three models of each SGTP to determine any unresolved secondary structures (i.e., α-helices).

The top candidate model structures were refined via minimization to remove steric clashes and optimization of the hydrogen-bond network by means of side chain sampling using the Schrödinger’s Maestro Protein Preparation Wizard [69]. Briefly, the Protein Preparation Wizard analyzes the structure to build a cluster of hydrogen bonds and with the highest degree of sampling, the algorithm then performs 100 000 Monte Carlo orientations for each cluster. Based on the electrostatic and geometric scoring functions, the algorithm then determines an optimized structure. Glucose and the other glucose transporter crystal structures (see below) were also prepared and optimized in this manner.

### Glucose induced-fit docking and migration

For the biophysical simulations, namely induced-fit docking and substrate migration, we used the state-of-the-art Protein Energy Landscape Exploration server (PELE) [70]. The PELE server provides ready-made scripts for substrate binding refinement (induced-fit docking) and unconstraint substrate binding site search (migration). The many uses of PELE can be accessed at https://pele.bsc.es/pele.wt and its algorithm is thoroughly explained in references [70, 71]. Briefly, the PELE algorithm performs three stages. First, localized substrate perturbations are performed. Protein perturbations of the α-carbon backbone are also performed using an anisotropic network model (ANM) [72]. Second, the amino acid side chains in proximity to the substrate are optimized using steric filters and a rotamer library [73]. Finally, a truncated Newton minimizer and a surface generalized Born implicit solvent for minimization [74] is used to achieve a local minimum after the initial perturbation. These three stages are performed for a number of steps and are analyzed in parallel with several computer-processing units. The results are a series of trajectories that represent protein side chain conformational changes and substrate migration. Based on the calculated energies of each step, a Monte Carlo Metropolis criterion implemented in PELE either accepts (if they are equal to and/or less than the initial energy) or rejects the steps (greater than the initial energy) [71]. The energy is calculated by using a standard force field to describe the potential energy of a particular molecular system, known as the optimized potentials for liquid simulations (OPLS-2005) [75].

Several parameters were optimized from the ready-made scripts provided by the PELE server to facilitate sampling. For the substrate binding refinement (induced-fit docking), (i) *steric_tr* (‘100’) and *tries* (‘25’) were reduced for the simulations to be less computationally expensive, (ii) translation (*tra_r* ‘2’) and rotation (*rot_r* ‘0.3’) of glucose were increased to permit more exploration of the active site, (iii) minimization radius (*mirad*) to allow minimization of the whole system, (iv) an ANM *type* to '4' was added and ANM *mode* changed to ‘5’ for favorable protein perturbations; and, (v) an increased number of steps (1000) was used for sufficient overall sampling. For the unconstrained substrate binding site search (migration), (i) *waitfor* was increased to ‘4’ to allow sufficient sampling of the active site, (ii) translation (*tra_r*) was reduced to ‘4’ to avoid large translation of glucose, (iii) an ANM *type* to '4' was added since the default ANM *mode* produced favorable protein perturbations; (iv) an increased number of steps (1000) was used for sufficient overall sampling; and, (v) a focused ANM (*anmrad* 15 Å) and minimization (*mirad* 10 Å) in proximity of the substrate was used for localized interpretation of glucose transport.

As proof of principle, we used two experimentally and structurally characterized glucose transporters: GLUT1 of *H. sapiens* [PDB: 4PYP] and XyIE of *E. coli* [PDB: 4GBZ]. The *xyz* coordinates of glucose from the crystal structure of *E. coli* XyIE were used as the starting position for all simulations (i.e., the active site of glucose transporters). The trajectories of glucose induced-fit (>2 000) and migration (>10 000) produced by the PELE algorithm were viewed and analyzed using the Visual Molecular Dynamics program (VMD) [76]. Clustering analyses of glucose migration was performed using VMD. The clustering parameters were set for ‘10’ clusters with a 10 Å cut-off distance for each cluster, since all simulations showed a concentrated glucose sampling of the active site (Additional file 2). SGTP1 was set with a 5 Å cut-off distance for each cluster since the majority of glucose exploration during the migration simulation was within the active site (Additional file 2). All plots were generated using GNUPLOT (http://gnuplot.sourceforge.net).

## Additional files

Additional file 1:
**Phylogeny of SGTP1, SGTP2, SGTP3 and SGTP4 homologs in** ***S. japonicum*** **and** ***S. haematobium.*** The figure shows a phylogenetic tree of the SGTP1, SGTP2, SGTP3 and SGTP4 homologs in *S. japonicum* (Sjap) and *S. haematobium* (Shae). The topology was obtained using the ML method. Numbers on internal branches are the bootstrap values. GenBank accession numbers of each sequence are included. (TIFF 16 kb) Additional file 2:
**Glucose migration from the active site of XylE, GLUT1 and** ***S. mansoni*** **glucose transporters.** The panels depict glucose migration Cartesian distances (given in Å) from the Tyr292/298 homologous residue (x-axis) and the active site (y-axis) for XylE, GLUT1, SGTP1, SGTP2, SGTP3 and SGTP4. (TIFF 1023 kb)

## Abbreviations

Å: angstrom
aLRT: approximate likelihood ratio test
bp: base pairs
cDNA: complementary deoxyribonucleic acid
LRT: likelihood ratio test
MEGA: molecular evolutionary genetics analysis
ML: maximum likelihood
mM: millimolar
MP: maximum parsimony
mRNA: messenger Ribonucleic acid
NJ: neighbor joining
PCR: polymerase chain reaction
PDB: protein data bank
PELE: protein energy landscape exploration
RACE PCR: rapid amplification of cDNA ends PCR
RMSD: root mean square deviation
RT-PCR: real-Time PCR
SH-like: Shimodaira-Hasegawa-like
