# Supplementary figures and images for: Fast evolutionary rates associated with functional loss in class I glucose transporters of Schistosoma mansoni
Source: BMC Genomics. 2015 Nov 19;16:980. doi: 10.1186/s12864-015-2144-6 (PMC4653847; doi:10.1186/s12864-015-2144-6)

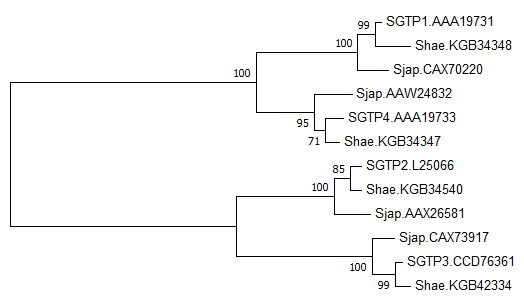

Supplement: Additional file 1: — Phylogeny of SGTP1, SGTP2, SGTP3 and SGTP4 homologs in S. japonicum and S. haematobium. The figure shows a phylogenetic tree of the SGTP1, SGTP2, SGTP3 and SGTP4 homologs in S. japonicum (Sjap) and S. haematobium (Shae). The topology was obtained using the ML method. Numbers on internal branches are the bootstrap values. GenBank accession numbers of each sequence are included. (TIFF 16 kb) [file 12864_2015_2144_MOESM1_ESM.tif]

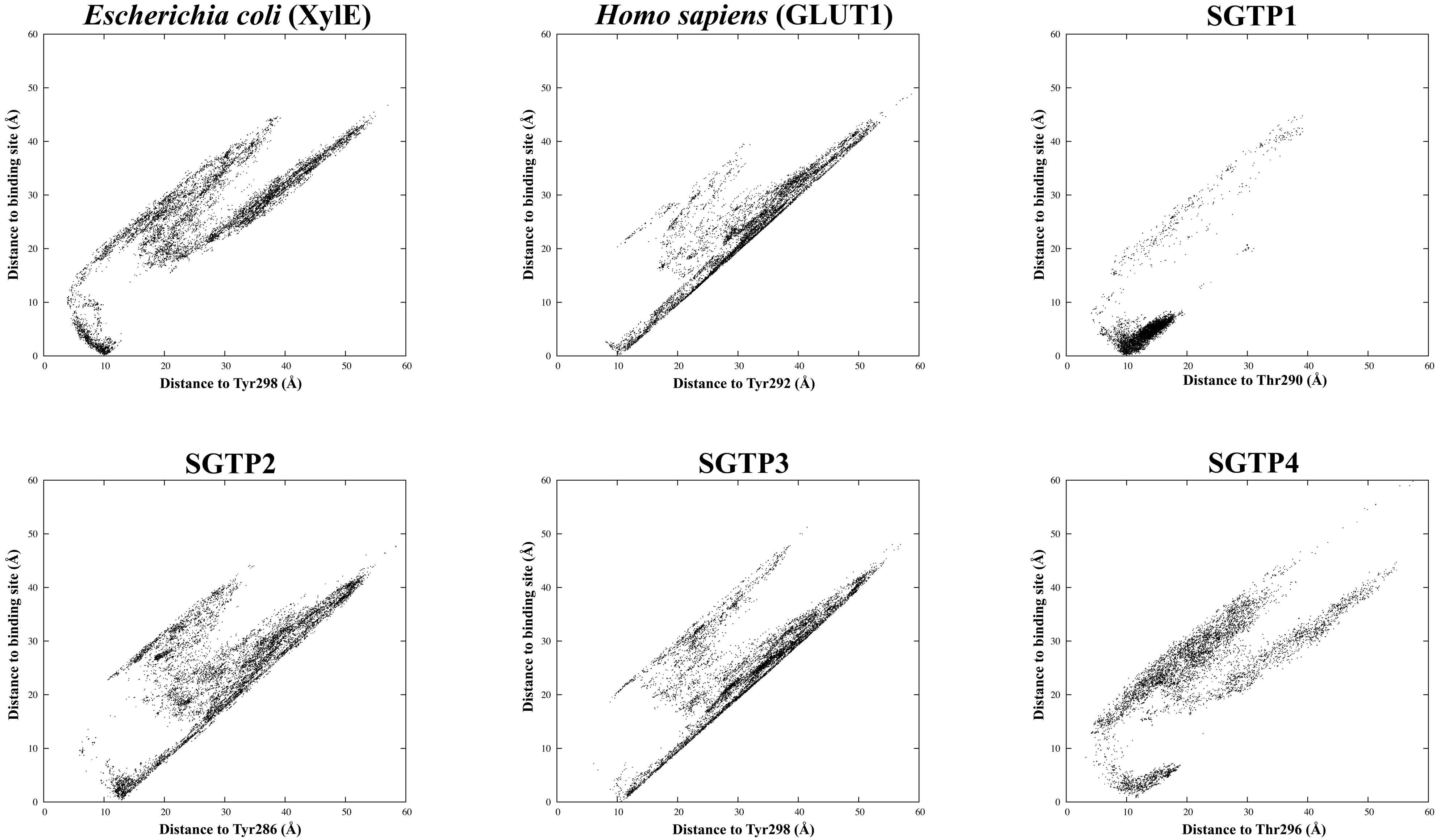

Supplement: Additional file 2: — Glucose migration from the active site of XylE, GLUT1 and S. mansoni glucose transporters. The panels depict glucose migration Cartesian distances (given in Å) from the Tyr292/298 homologous residue (x-axis) and the active site (y-axis) for XylE, GLUT1, SGTP1, SGTP2, SGTP3 and SGTP4. (TIFF 1023 kb) [file 12864_2015_2144_MOESM2_ESM.tif]
